# Intravenous Injections of Quinine for the Treatment of Primary Paludism

**Published:** 1918

**Authors:** 


***Intravenous Injections of Quinine for the Treatment of Primary Paludism*** By P. Carnot and A. de Kerdrel. Trans. and Abs. from the *Paris Médical*, Vol. XXIII, p. 2.

The authors state that in spite of strong and prolonged dosing with quinine, administered through the digestive and interstitial tracts, many of the cases of paludism evacuated from the Orient to France remain badly infected.

The method of Bacelli, for complications during the first month, has given better results than any other means of absorption.

*Technique of Intravenous Injections of Quinine.* Urethane quinine is practically the only solution employed. Used interstitially, this is less painful, less caustic, more quickly absorbed than the other solutions of quinine.

Ampoules are prepared containing the following ingredients:

| | |
|---|---|
| Basic chloro-hydrate of quinine. . . . . . . . . | 0.40 gr. |
| Uretane. . . . . . . . . . . . . . . . . . . . . | 0.20 " |
| Distilled water. . . . . . . . . . . . . . . . . | 1.00 cc. |

For intravenous injections each ampoule is diluted in 22 cc. of physiological saline, bringing the total of quinine in the solution down to 2 per cent. In serious cases good results have been obtained by injecting with the diluted quinine solution, a certain amount of physiological serum. The total of dilution is reduced to 1.6 or 3.2 per cent.

Intravenous injections of quinine must be made slowly at the rate of 20 cc. of saline plus 0.4 gr. of urethane quinine in 5 minutes.

*Pernicious Complications.* Serious complications resulting from severe infection may severely affect the nervous system and the suprarenal, hepatic, or renal glands. These are sometimes generalized and sometimes confined to the nervous system, the liver, and the kidneys.

*Generalized forms.* Complications resembling typhoid. The dynamic attack is not the first manifestation of paludism. Earlier signs determine the diagnosis. Temperature, taken every three hours, shows considerable variation. The spleen is usually much more enlarged and painful than in typhoid. Blood examination by smears reveals numerous merozoites and crescents. If this result is not obtained from the peripheral blood, it may be observed in the visceral blood. It is an indication of serious paludism.

A large intravenous injection should immediately be given. Two injections of 0.40 gr. with an interval of a few hours may be preferred to one large one of 0.80 gr. diluted in 20 cc. of saline.

In such cases, the best results have been obtained.

*Nervous manifestations* (delirious, algid, comatose, hemiplegic, epileptic, etc.) Serious and alarming symptoms often result from the generalized infection or its localization in the nervous system or meninges. Death appears to be imminent. Intravenous injections of quinine is indicated in such cases.

